# B-cell lymphoma with cytokine storm in serosal effusion: A case report and literature review

**DOI:** 10.1097/MD.0000000000037400

**Published:** 2024-03-08

**Authors:** Xiaoli Zhang, Xueze Shi, Xixi Liu, Chencheng Li, Zuqiong Xu, Xingbin Dai, Bangyun Ma, Xuejun Zhu

**Affiliations:** a Nanjing University of Chinese Medicine, Nanjing, China; b Hematology Department of Affiliated Hospital of Nanjing University of Chinese Medicine, Nanjing, China.

**Keywords:** B-cell lymphoma, multiple serous cavity effusion, regional cytokine storms

## Abstract

**Rationale:**

Cytokine storm is now considered to be a systemic inflammatory response, but local cytokine storm may exist in systemic diseases of the blood system. Monitoring of regional cytokine storm is an important clue for the diagnosis of systemic diseases.

**Patient concerns:**

A 72-years-old male presented to our hospital with multiple serosal effusion without solid mass or enlarged lymph nodes. We found that the level of cytokines in ascites was tens to hundreds of times higher than that in plasma, mainly IL-6 and IL-8.

**Diagnoses:**

The patient was diagnosed with multiple serous effusion, hemophagocytic syndrome, B-cell lymphoma, Epstein–Barr virus infection, and hypoproteinemia.

**Interventions:**

During hospitalization, the patient was treated with 5 courses of R-CVEP therapy and supportive treatment.

**Outcomes:**

After the first R-CVEP regimen, the patient’s condition was evaluated as follows: hemophagocytic syndrome improved: no fever; Serum triglyceride 2.36 mmol/L; Ferritin 70.70 ng/L; no hemophagocyte was found in the bone marrow; the lymphoma was relieved, ascites disappeared, and bone marrow cytology showed: the bone marrow hyperplasia was reduced, and small platelet clusters were easily seen. Bone marrow flow cytometry showed that lymphocytes accounted for 13.7%, T cells increased for 85.7%, CD4/CD8 = 0.63, B cells decreased significantly for 0.27%, and NK cells accounted for 10.2%. Blood routine returned to normal: WBC 5.27 × 10^9^/L, HB 128 g/L, PLT 129 × 10^9^/L; Epstein–Barr virus DNA < 5.2E + 02 copies/mL; correction of hypoproteinemia: albumin 39.7 g/L.

**Lessons:**

Cytokines in ascites are significantly higher than those in plasma by tens to hundreds of times, suggesting that “regional cytokine storms” may cause serosal effusion.

## 1. Introduction

Polyserous effusion refers to the simultaneous effusion of 2 or more serous cavities in the thoracic cavity, pericardial cavity, and abdominal cavity, which has become a common and difficult clinical disease at present, and is a complication of a variety of benign and malignant diseases, such as infection, hypoalbuminemia, malignant tumor, connective tissue disease, cirrhosis, heart failure, etc, most commonly seen in malignant tumor.^[1]^ More than one-third of polyserous cavity effusions are of unknown cause.^[2]^ Studies have found that 20% to 30% of non-Hodgkin lymphoma invading the serous membrane can cause pleural effusion and pericardial effusion,^[3]^ among which pleural effusion is the most common.^[4]^ We report a case of B-cell lymphoma with multiple serosal effusion as the main clinical manifestations, no solid mass and enlarged lymph nodes, combined with Epstein–Barr virus (EBV) infection and hemophagocytic syndrome. Cytokine levels (mainly IL-6 and IL-8) were consistently detected in ascites, which were more than 10 times higher than that of plasma. Therefore, it was analyzed that the cause of the fluid accumulation in the serosal cavity in this case may be the generation of “regional cytokine storm,” and the literature was reviewed according to the case.

## 2. Case report

A 72-years-old male suffered from fatigue, chest tightness, and tightness in early April 2022, accompanied by poor appetite, diarrhea, and mild edema of both lower extremities, and abdominal B-ultrasound indicated hydrops in the chest and abdomen. He was hospitalized in a local hospital, and the auxiliary examination indicated “pulmonary infection, polyserous effusion, hypoproteinemia, possible cardiac insufficiency, and thrombocytopenia.” During the course of the disease, high fever (peak temperature >38.5°C) occurred. After treatment with methylprednisolone, antiinfection, pleural effusion puncture and drainage, and albumin supplementation, diarrhea improved, peak temperature decreased, and residual symptoms did not improve significantly. Hydrothorax and abdomen are still obvious. He went to our hospital for treatment on April 27, 2022, and was admitted to the outpatient department with “multiple serous cavity effusion to be investigated.” Physical examination after admission: poor mental condition, mild anemia appearance, weakened respiratory sound in the right lung, no obvious dry and wet rales, crepitus, drainage tube in the chest wall of the right lower lung. Abdominal distension, no tenderness and rebound pain, mobile dullness is positive, no bowel sound, mild edema of both lower limbs, the rest of the physical examination was normal.

**Figure 1.**
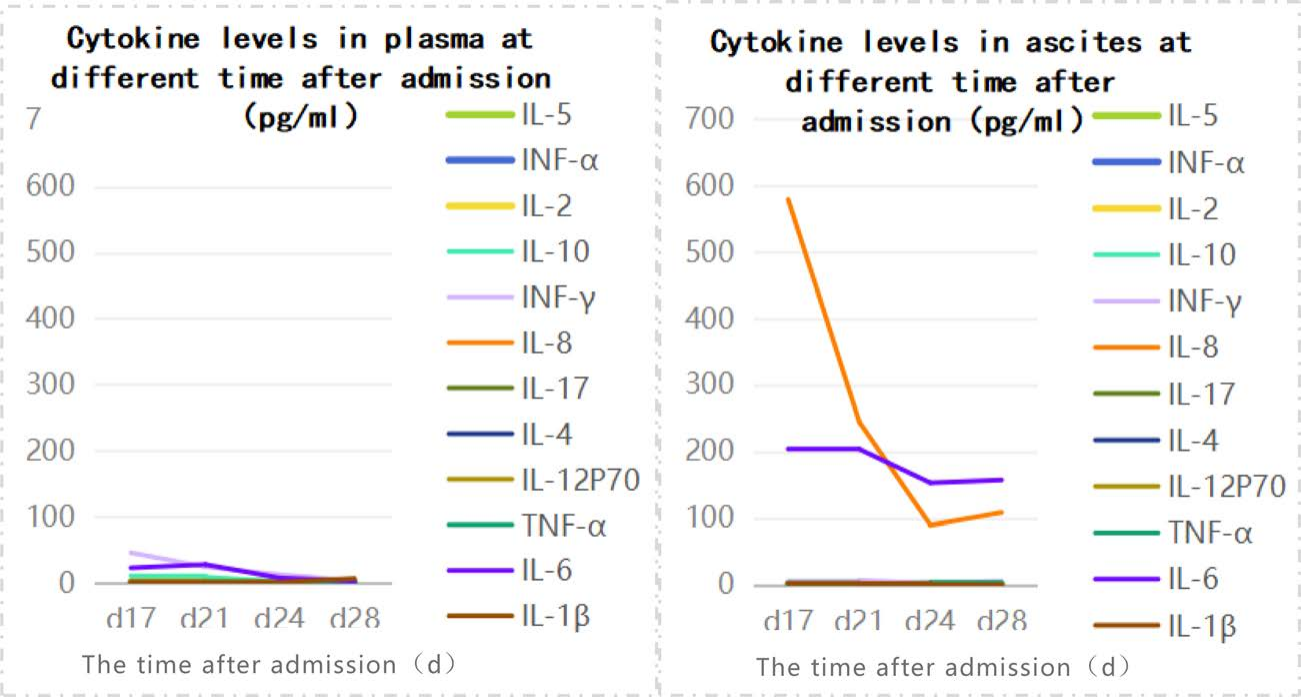
Cytokine levels in plasma and ascites at different time after admission (pg/mL).

Relevant examinations were completed after admission. Blood routine + hypersensitive C-reactive protein: CRP 13.83 mg/L, WBC 8.49 × 10^9^/L, HB 117 g/L, PLT 36 × 10^9^/L; EBV DNA: 2.05E + 03; Serum triglyceride: 3.94 mmol/L; Ferritin >1500 ng/mL; Soluble CD25 4554.34 U/mL; Chromosome: 45, XY, i(6)(P10),? del(10)(q26), add(11)(q23), der (15; 21), (q10; q10), i(18)(q10), x2[20]; IG-κ gene rearrangement is positive, the rest were negative; T cell receptor gene rearrangement is negative. The copy number of lymphocyte subsets infected with EBV was detected by magnetic bead sorting: B cells 1.3E + 04, NK/NK-T cells 1.3E + 03, CD4 + T cells 8.9E + 02, CD8 + T cells were not detected. IL-6 was significantly increased in ascites and slightly increased in plasma (Fig. 1). There were no obvious abnormalities in the routine and biochemical aspects of thoracoabdominal fluid. Common cytological smear of ascites: atypical cells were found, and no malignant evidence was found by immunohistochemistry. Immunotyping of leukemia (ascites): ascites mainly consisted of lymphocytes (82.1%), T cells increased (94.3%), CD4/CD8 = 1.08, B cells decreased (3.20%), NK cells decreased (1.15%), and NK-T cells (10.0%). Morphological analysis of bone marrow cells: bone marrow hyperplasia was active and abnormal cells accounted for 5.0%. Hemophagy is easy to see. Megakaryosis is active, plate production is greatly reduced, and platelet dispersion is rare. Leukemia immune typing (bone marrow): the proportion of bone marrow lymphocytes increased by 60.5%, in which abnormally increased B cells were the main cells. They accounted for 43.4% of lymphocytes and 24.9% of total white blood cells, expressing CD5, CD19, CD20, CD22, CD23, and FMC7, partially/weakly expressing CD38, CD11c, CD200, and CD81, not expressing CD10, CD25, and other antigens, the expression of light chains is restrictive. Bone marrow puncture pathology: hematopoietic tissue hyperplasia was acceptable. Interstitial lymphocytes proliferate. Immunohistochemical results: CD3 (scattered +), CD5 (scattered +), CD20 (individual cell +), CD79a (individual cell +), Ki67 (about 20%+), CD10 (−), BCL-6 (−), BCL-2 (scattered +), CyclinD1 (−), CD23 (−), LEF-1 (−), CD43 (partial +), CD38 (scattered +), CD138 (scattered +), κ (+), λ (+). Full abdominal CT: abdominal pelvic effusion, slightly larger bilateral inguinal lymph nodes, small pericardial effusion, multiple hepatic cysts, atherosclerosis, and possible abdominal wall thrombosis of the right common iliac artery. Combined with the patient’s history and auxiliary examination results, the diagnosis was as follows: multiple serous effusion; hemophagocytic syndrome; B-cell lymphoma; EBV infection; and hypoproteinemia.

**Figure 2.**
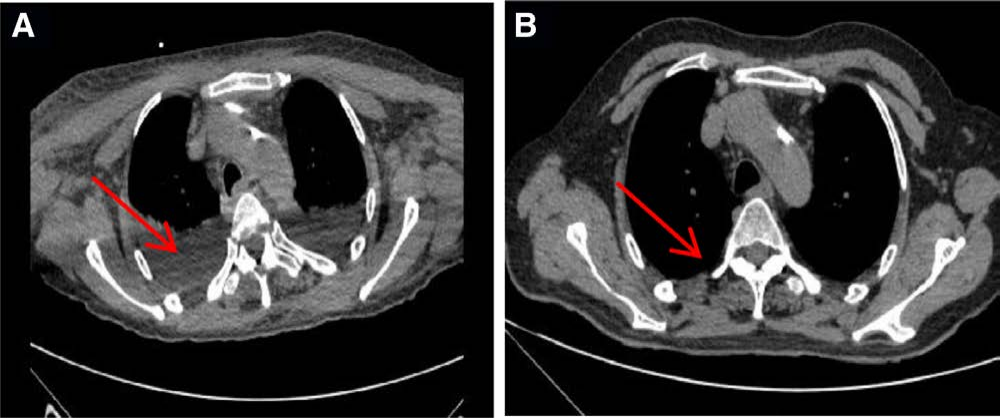
Changes of the patient’s pleural effusion before and after treatment (A: pleural effusion before treatment; B: pleural effusion did not show after treatment).

**Figure 3.**
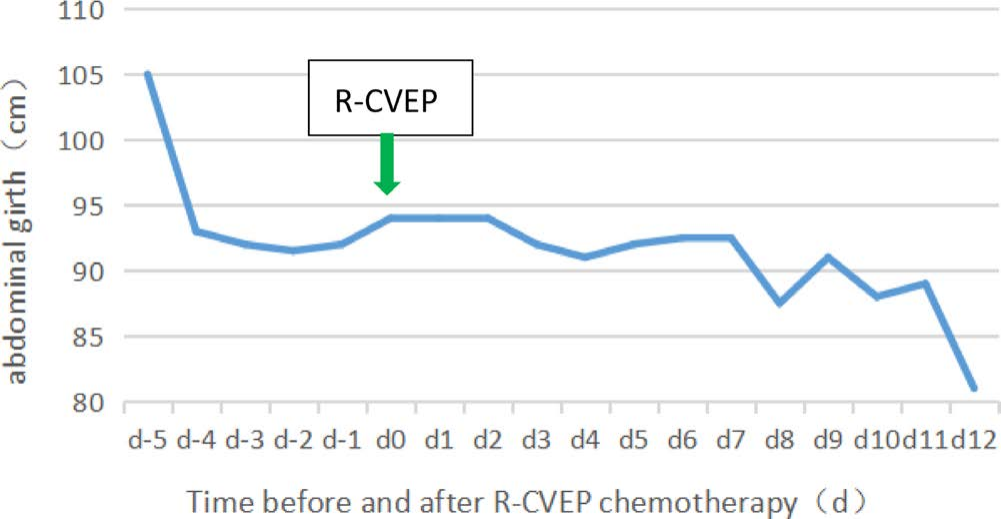
Changes of abdominal girth before and after R-CVEP chemotherapy. R-CVEP:Rituximab 0.6 g intravenous drip (d0 = 0.1 g, d1 = 0.5 g), cyclophosphamide 0.4 g intravenous drip d1-2, etoposide softgel 50 mg orally d1-5, vindesine 2 mg intravenous drip d1, dexamethasone 10 mg intravenous drip d1-5.

In terms of treatment, on May 12, intravenous propyl bulb 20 g qd was injected for 5 days and dexamethasone 10 mg qd was administered for 9 days to control hemophagocytic syndrome, and after the diagnosis of B-cell lymphoma was confirmed, R-CVEP chemotherapy began on May 20, 2022: Rituximab 0.6 g intravenous drip (d0 = 0.1 g, d1 = 0.5 g), cyclophosphamide 0.4 g intravenous drip d1-2, etoposide softgel 50 mg orally d1-5, vindesine 2 mg intravenous drip d1, dexamethasone injection 10 mg intravenous drip d1-5. The chemotherapy process was smooth, during which the patient had abnormal liver function and low fibrinogen, continued liver preservation, and other treatments, supplemented by cryoprecipitation infusion, platelet, and diuresis, the patient’s ascites gradually decreased (Fig. 2), abdominal circumference decreased (Fig. 3), and drainage tube was removed. After chemotherapy, the patient developed myelosuppression, leukopenia, and granulocyte colony-stimulating factor to stimulate the growth of granulocyte system, and anti-infectional treatment with biapenem, improved and was discharged. Then the patient underwent 5-cycle R-CVEP regimen. After the first R-CVEP regimen, the patient’s condition was evaluated as follows: hemophagocytic syndrome improved: no fever; Serum triglyceride 2.36 mmol/L; Ferritin 70.70 ng/L; no hemophagocyte was found in the bone marrow; the lymphoma was relieved, ascites disappeared, and bone marrow cytology showed: the bone marrow hyperplasia was reduced, and small platelet clusters were easily seen. Bone marrow flow cytometry showed that lymphocytes accounted for 13.7%, T cells increased for 85.7%, CD4/CD8 = 0.63, B cells decreased significantly for 0.27%, and NK cells accounted for 10.2%. Blood routine returned to normal: WBC 5.27 × 10^9^/L, HB 128 g/L, PLT 129 × 10^9^/L; EBV DNA < 5.2E + 02 copies/mL; correction of hypoproteinemia: albumin 39.7 g/L.

## 3. Discussion

### 3.1. Differential diagnosis

This disease should be distinguished from primary effusion lymphoma (PEL): similar to this case, both patients may have multiple serosal effusion, most of which are not accompanied by mass.^[5]^ Symptoms can include difficulty breathing and diarrhea, EB virus infection occurs in 80% of patients with PEL.^[6]^ The main differences between PEL and this patient are as follows: PEL is more common in young and middle-aged gay or bisexual men infected with human immunodeficiency virus, and is closely related to human herpesvirus-8. The patient in this case was an elderly man, and neither HIV nor to human herpesvirus-8 was detected; the serosal effusion in PEL is malignant exudate. The diagnosis of the disease is usually determined by the histopathological immunohistochemistry of the effusion (tumor cells are found only in the body cavity^[7]^). However, the effusion in this case was leaking fluid, and no evidence of malignancy was found by pathological immunohistochemistry; in previous case reports of PEL, rituximab was not effective because PEL does not express B-cell antigen.^[7,8]^ In this case, the bone marrow pathology indicated CD20+, and rituximab had a better effect. The median survival of PEL is <6 months, and the 1-year survival rate is about 30%,^[9]^ the patient is still alive and in good condition.

### 3.2. Cause analysis of multiple serous cavity effusion in this case

Polyserous effusion has become a common and difficult disease in clinical practice. It is a complication of a variety of benign and malignant diseases, such as infection, hypoalbuminemia, malignant tumor, connective tissue disease, cirrhosis, heart failure, etc. The most common occurrence is malignant tumor. More than 1/3 polyserous effusion has unknown cause. Lymphoma-associated serous effusion may be primary or secondary. Secondary serous effusion is often a complication of advanced disease and rarely an initial manifestation,^[10]^ while the primary effusion originates in the body cavity and there is no detectable mass.^[4,5,11,12]^ At the onset of the disease, the main manifestation of the patient is multiple serous effusion, no solid mass and enlarged lymph nodes, and it is easy to be misdiagnosed as primary exudative lymphoma, and the diagnosis is eventually excluded by ascites and bone marrow related examination. The formation of multiserosal effusion in this patient can be explained from the perspective of “regional cytokine storm.” Cytokine storm can be caused by various pathogens such as EBV, autoimmune diseases, malignant diseases, genetic diseases, or some therapeutic interventions. Its main feature is that immune cells are over-activated and a large number of cytokines such as IL-6 are released, causing serious damage to the body.^[13]^ IL-6 is produced by immune and nonimmune cells in multiple organ systems and sends signals primarily through 2 pathways (classical cis-signaling and trans-signaling), where activation of cis-signaling leading to pleiotropic effects on the immune system may be the mechanism for cytokine storm generation.^[14]^ Cytokine storm is a systemic inflammatory response in which elevated cytokines are detected almost exclusively in the bloodstream and rarely in the body cavity.^[13]^ In this case, the cytokine elevation was mainly in the abdominal cavity. We continuously measured the cytokine levels in plasma and ascites of patients on day 17, day 21, day 24, and day 28 after admission, and found that the cytokine levels in ascites increased significantly, mainly IL-6 and IL-8. IL-6 levels in ascites were 9 times, 7 times, 19 times, and 47 times of IL-6 levels in plasma, respectively, and IL-8 levels in ascites were more than 400 times of IL-8 levels in plasma (Fig. 1). Therefore, we speculated that the formation of multi-serosal effusion in this patient was due to the activation of immune cells after EBV infection, which triggered a “regional cytokine storm,” resulting in a large release of cytokines mainly IL-6 and IL-8, and then resulting in capillary leakage and serosal effusion. The difference between ascites and plasma cytokines in this case reminds us of the necessity of detecting ascites cytokines, which has certain significance for monitoring and guiding treatment of local cytokine storms.

## 4. Conclusion

B-cell lymphoma with multiple serosal effusion as its main manifestation is rare. In this case, the patient is combined with EBV infection and hemophagocytic syndrome, which is challenging for accurate diagnosis and differential diagnosis. We need to distinguish patients from other malignant tumors and implement appropriate treatment for B-cell lymphoma as early as possible through comprehensive judgment of bone marrow morphology, pathology, immune typing, chest and abdominal fluid examination, and virology examination. At the same time, based on the differences in the levels of cytokines in ascites and plasma of this patient, we speculated that there may be a “regional cytokine storm” leading to the formation of polyserous effusion. The deficiency of this paper is that there is a lack of extensive literature support on “regional cytokine storm,” and the mechanism of EBV causing local cytokine storm needs further research to verify.

## Author contributions

**Writing—original draft:** Xiaoli Zhang, Xueze Shi.

**Data curation:** Xixi Liu, Chencheng Li.

**Conceptualization:** Zuqiong Xu, Xingbin Dai.

**Writing—review & editing:** Bangyun Ma, Xuejun Zhu.
